# On the Mutual Action of the Elements of Soluble Salts without and within the Animal Economy

**Published:** 1866-11

**Authors:** M. Melsens


### Miscellaneous.

### On the Mutual Action of the Elements of Soluble Salts without and within the Animal Economy.

BY M. MELSENS.

The experiments already made by the author, he considers, justify him in formulating the following proposition:

*Two soluble salts which are without apparent mutual action, and which may be given separately to animals without producing any disturbance in the body, may, when administered together, act as a poison.*

Facts of this kind, proved by experiment, have a great importance, both from physiological and therapeutical points of view.

The two salts more especially experimented with by the author, are chlorate of potash and iodide of potassium. These two salts in solution together crystalize separately under whatever conditions they may be placed. Their mixture in solution in equivalent proportions undergoes no mutual decomposition either at the ordinary temperature, on boiling, or under the pressure of 10 atmospheres at 185° C. It can be proved by means of sulphydric acid that no iodate of potash is formed.

But when the two salts are heated together in the dry state, decomposition takes place at the point of fusion, and iodate of potash is formed.

When a certain quantity of mineral acid is added to a mixed solution of the two salts, iodine is set at liberty, and the solution behaves towards sulphydric acid as though iodic acid had been produced.

When the mixed solutions are submitted to electrolysis, hydrogen is disengaged at the positive pole, and the liquor appears to contain both iodide and iodate.*

We come next to the effects of the before-mentioned salts on animals. ¶ Seven grains of chlorate of potash were given to a bitch

* To avoid the action of chlorine, iodine, and oxygen, the author employed retort coke as the positive electrode. The carbon was previously treated with aqua regia, and then ignited in a current of chlorine. The carbon employed in the above experiment was disaggregated, and in part burnt to carbonic oxide and acid, and at the same time a soluble black carbonaceous matter was obtained, similar to the ulmic product which the author obtained by the action of chlorine on the carbon contained in the lungs in *melanosis*.

weighing eleven kilos, every day for a month; the animal did not at all suffer. Afterwards five grammes of iodide of potassium were given daily for the same period. The animal suffered a little during the first days, but at the end of the month was perfectly well.

If, on the contrary, we administer to a dog daily seven grammes of a mixture of iodide of potassium and chlorate of potash in equivalent proportions, the animal languishes and dies about the twenty-fifth or twenty-eighth day. On commencing the experiment one dog weighed 16.5 kilos; at the moment of its death it weighed only 11.5 kilos. The experiment repeated on several dogs gave similar results. Death often supervened about the fifth day.

Post mortem examinations revealed changes similar to those observed by the author when iodate of potash was administered, especially in the liver and intestines, but it is necessary to make a series of comparative experiments with the iodate, free iodine, and mixtures of the two salts.

The author has shown in previous memoirs that the iodate of potash acts as a poison. This salt, given in doses of one or two grammes daily, will kill a small dog in a few days. A mixture of the two salts can not be so active as the iodate, since both unchanged iodide and chlorate may be found in the urine. The author is therefore brought to the conclusion that the mutual action of the two salts in the economy takes place with the greatest facility. It may be supposed that the acids of the stomach and the electrolytic actions which take place in the organism play an important part in bringing about this decomposition. But beyond all hypothesis it is necessary to admit that changes take place in the animal system which cannot be realized in the laboratory under ordinary conditions, or with the assistance of a high temperature, strong acids, or even the electric current.—*Lond. Chem. News*, August 17, 1866.

---

M. Rossi writes to the Roman "Correspondenza Scientifica," stating that by the use of the Erythroxyion coca of Peru, men may live in robust health for several days without food. M. Rossi declares that after taking a decoction of the leaves of the plant he felt neither hunger nor thirst for forty-eight hours.

